# Higher risk for influenza‐associated pulmonary aspergillosis (IAPA) in asthmatic patients: A Swiss multicenter cohort study on IAPA in critically ill influenza patients

**DOI:** 10.1111/irv.13059

**Published:** 2022-11-16

**Authors:** Frederike Waldeck, Filippo Boroli, Sandra Zingg, Laura N. Walti, Pedro David Wendel‐Garcia, Anna Conen, Jean‐Luc Pagani, Katia Boggian, Madeleine Schnorf, Martin Siegemund, Samia Abed‐Maillard, Marc Michot, Yok‐Ai Que, Veronika Bättig, Noémie Suh, Gian‐Reto Kleger, Werner C. Albrich

**Affiliations:** ^1^ Division of Infectious Diseases and Microbiology University Hospital Schleswig Holstein, Campus Lübeck Lübeck Germany; ^2^ Division of Intensive Care Geneva University Hospitals Geneva Switzerland; ^3^ Division of Infectious Diseases and Hospital Epidemiology University Hospital Basel Basel Switzerland; ^4^ Division of Infectious Diseases, Inselspital Bern University Hospital Bern Switzerland; ^5^ Institute of Intensive Care Medicine University Hospital Zurich Zürich Switzerland; ^6^ Division of Infectious Diseases and Infection Prevention Cantonal Hospital Aarau Aarau Switzerland; ^7^ Division of Intensive Care University Hospital Lausanne Lausanne Switzerland; ^8^ Division of Infectious Diseases and Hospital Epidemiology Cantonal Hospital St. Gallen St. Gallen Switzerland; ^9^ Intensive Care Unit, Department of Acute Care University Hospital Basel Basel Switzerland; ^10^ Division of Intensive Care Cantonal Hospital Thun Thun Switzerland; ^11^ Division of Intensive Care, Inselspital Bern University Hospital, University of Bern Bern Switzerland; ^12^ Division of Intensive Care Cantonal Hospital St. Gallen St. Gallen Switzerland

**Keywords:** asthma, influenza, influenza‐associated aspergillosis, intensive care medicine, invasive aspergillosis

## Abstract

**Background:**

Influenza‐associated pulmonary aspergillosis (IAPA) is an important complication of severe influenza with high morbidity and mortality.

**Methods:**

We conducted a retrospective multicenter study in tertiary hospitals in Switzerland during 2017/2018 and 2019/2020 influenza seasons. All adults with PCR‐confirmed influenza infection and treatment on intensive‐care unit (ICU) for >24 h were included. IAPA was diagnosed according to previously published clinical, radiological, and microbiological criteria. We assessed risk factors for IAPA and predictors for poor outcome, which was a composite of in‐hospital mortality, ICU length of stay ≥7 days, mechanical ventilation ≥7 days, or extracorporeal membrane oxygenation.

**Results:**

One hundred fifty‐eight patients (median age 64 years, 45% females) with influenza were included, of which 17 (10.8%) had IAPA. Asthma was more common in IAPA patients (17% vs. 4% in non‐IAPA, P = 0.05). Asthma (OR 12.0 [95% CI 2.1–67.2]) and days of mechanical ventilation (OR 1.1 [1.1–1.2]) were associated with IAPA. IAPA patients frequently required organ supportive therapies including mechanical ventilation (88% in IAPA vs. 53% in non‐IAPA, P = 0.001) and vasoactive support (75% vs. 45%, P = 0.03) and had more complications including ARDS (53% vs. 26%, P = 0.04), respiratory bacterial infections (65% vs. 37%, P = 0.04), and higher ICU‐mortality (35% vs. 16.4%, P = 0.05). IAPA (OR 28.8 [3.3–253.4]), influenza A (OR 3.3 [1.4–7.8]), and higher SAPS II score (OR 1.07 [1.05–1.10]) were independent predictors of poor outcome.

**Interpretation:**

High clinical suspicion, early diagnostics, and therapy are indicated in IAPA because of high morbidity and mortality. Asthma is likely an underappreciated risk factor for IAPA.

**Abbreviations**
ARDS: acute respiratory distress syndrome
BAL: bronchoalveolar lavage
COPD: chronic obstructive pulmonary disease
COVID‐19: coronavirus infection 2019, SARS‐Cov‐2
CT‐scan: computer tomography
ECMO: extracorporeal membrane oxygenation
FUNGINOS: Swiss network on fungal diseases
GM: galactomannan
IAPA: influenza‐associated pulmonary aspergillosis
ICU: intensive care unit
LOS: length of stay
IQR: interquartile range
non‐IAPA: influenza patients without influenza‐associated pulmonary aspergillosis
OR: odds ratio
SAPS II: simplified acute physiology score
TS: tracheal secretion
95% CI: 95% confidence interval

## 1 INTRODUCTION

Influenza is a known risk factor for invasive pulmonary aspergillosis,^1^ as are other viral respiratory infections including SARS‐Cov‐2 (COVID‐19),^2^ parainfluenza, and respiratory syncytial virus.^3^ Since its first case description in 1952,^4^ influenza‐associated pulmonary aspergillosis (IAPA) is increasingly recognized as a severe complication in critically ill influenza patients.^5^, ^6^, ^7^, ^8^ IAPA incidence ranges from 10% to 32% of influenza patients admitted to ICU.^1^, ^9^, ^10^ Differences in prevalence might be related to different awareness and screening practices.^11^ Risk factors for developing IAPA include male sex, smoking, chronic lung disease, influenza A, solid organ transplant, hematologic malignancy, and treatment with corticosteroids within 28 days prior to influenza infection but are otherwise poorly defined.^1^, ^26^ IAPA carries a high mortality of 30–60%^1^, ^5^, ^6^, ^12^, ^13^ and commonly results in need of organ supportive therapies.^14^ Optimal diagnostic and preventive strategies are unclear.^15^

During the 2017/2018 season, we identified IAPA in 11% of critically ill influenza patients in two Swiss centers, which was associated with high risk of complications and mortality.^14^ In the current study, we aimed to analyze the epidemiology and clinical outcome of IAPA in a multicenter study of Swiss ICUs combining our 2017/2018 data with new data from 2019/2020.

## 2 STUDY DESIGN AND METHODS

**FIGURE 1:**
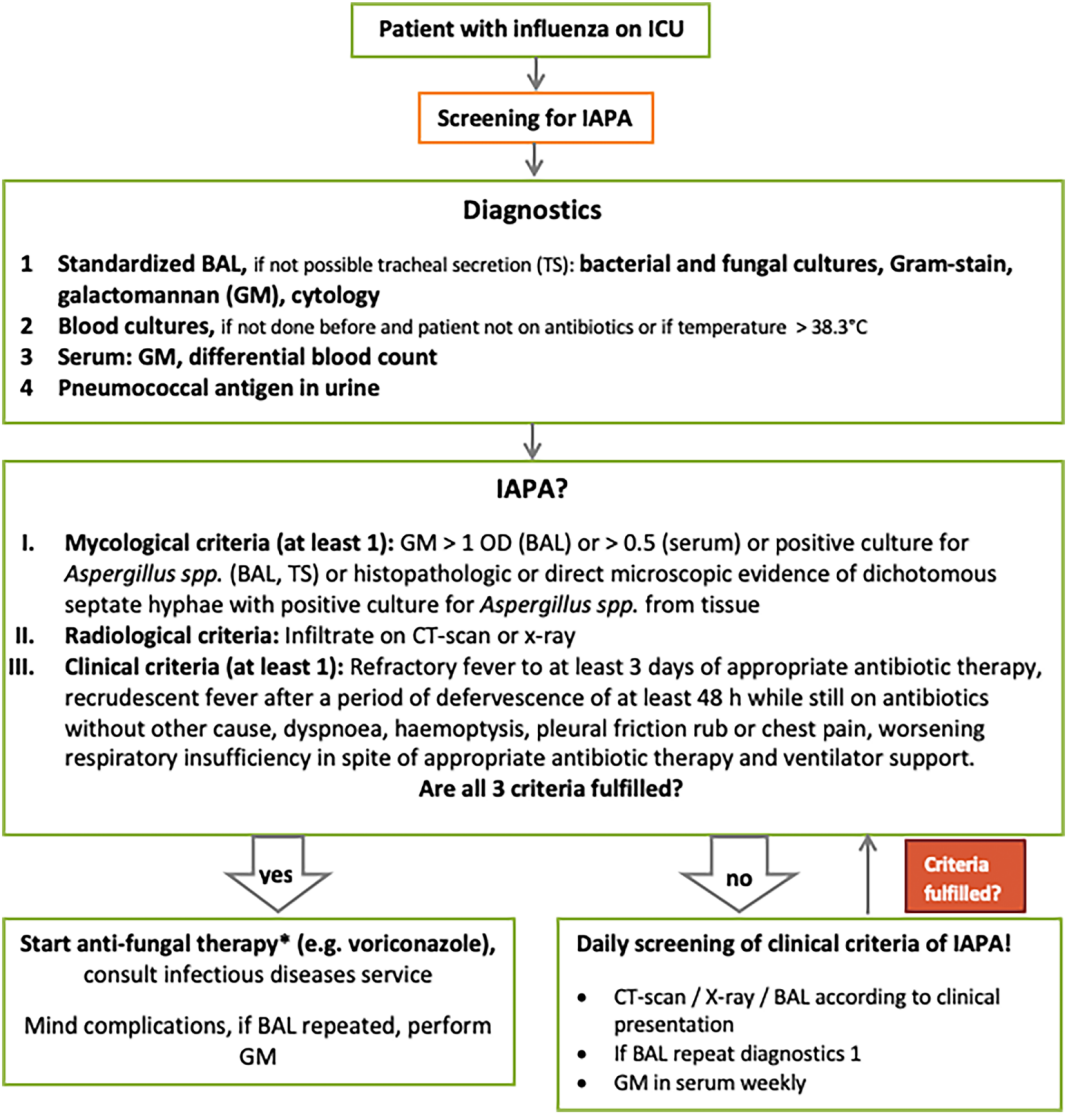
Screening algorithm for IAPA in critically ill patients with influenza on the ICU. *If critically ill and unstable patient with suitable clinical presentation of IAPA start preemptive therapy before microbiological results are obtained. Determine GM before starting antifungal treatment because of rapid decrease under therapy. Determine voriconazole trough level after 5–7 days (aim at 1–5 mg/L). Alternative treatment: liposomal amphotericin B. IAPA = influenza‐associated pulmonary aspergillosis, BAL = bronchoalveolar lavage, TS = tracheal secretion

We performed a multicenter cohort study in ICUs of seven tertiary care hospitals in Switzerland during the 2019/2020 influenza season. Participating ICUs received an educational training on influenza and IAPA in fall 2019 and a screening algorithm for IAPA was implemented (Figure 1) in order to increase awareness of this entity and homogenize diagnostics and treatment strategies. We proposed to screen all patients with influenza infection systematically for IAPA on admission to the ICU using fungal cultures from bronchoalveolar lavage (BAL) or tracheal secretion (TS), if BAL was not possible, and galactomannan (GM) from BAL or serum. A positive GM was defined as >0.5 in serum and >1.0 in BAL. Patients were assessed daily for new clinical signs of IAPA, which triggered microbiological sampling for aspergillosis from BAL (or TS) and serum. IAPA was diagnosed according to diagnostic criteria by Schauwvlieghe *et al* and Blot *et al*.^1^, ^16^ Anti‐mold treatment was initiated as soon as criteria of IAPA were fulfilled or pre‐emptively if patients were unstable and had suspicion of IAPA (Figure 1). All data were retrospectively collected from electronic medical records. Our inclusion criteria and definitions were reported previously.^14^

We combined data on influenza patients from seven ICUs during the 2019/2020 influenza season with those from the 2017/2018 influenza season of two tertiary care hospitals (University Hospital Geneva, Cantonal Hospital St. Gallen). The primary outcome was to find risk factors for IAPA and secondary outcomes were to define predictors for poor outcome, which was a composite of in‐hospital mortality, ICU length of stay (LOS) ≥ 7 days, mechanical ventilation ≥ 7 days, or extracorporeal membrane oxygenation (ECMO).

The study was approved by the local ethics committees (EKOS 2018‐01994 and 2019‐02173). Funding was provided by the research funding of the Cantonal Hospital of St. Gallen and the Swiss network on fungal diseases (FUNGINOS).

### 2.1 Statistical analysis

No missing data were observed in 158 data sets. Continuous variables were not categorized. Continuous variables were assessed by Wilcoxon ranksum test, categorical values by Fisher's exact test. Multivariable logistic regression was performed to assess risk factors for IAPA and the composite poor outcome. To prevent multicollinearity, we first removed variables which were obviously (per definition, or clinically) related to the relevant outcomes (antifungal therapy for IAA, and intubation, renal replacement therapy, ARDS, bacterial superinfection, and delirium for bad outcome). The last two variables, for example, are known to be associated with ICU‐LOS.

Variables with very low numbers were also removed from the model (i.e., neutropenia and solid organ transplantation). Restricted cubic splines were used for modelling continuous variables. We performed a stepwise backward elimination procedure. Collinearity was tested with variance inflation factor and variables that were highly collinear were eliminated. Bootstrapping procedures were used to check validation and calibration of the model. Kaplan–Meier curves were analyzed for assessment of duration of ICU stay. Univariate analysis of LOS‐ICU was performed by the logrank test. All statistical analyses were performed by R 4.0.2 (2017, R Foundation for Statistical Computing, Vienna, Austria).

## 3 RESULTS

We included 158 influenza patients (81 [51%] from the 2017/2018 and 77 [49%] from the 2019/2020 influenza season). Seventeen patients (10.8%) were diagnosed with IAPA. We did not observe a different proportion of IAPA over the two influenza seasons (2017/18: 9 cases [11.1%], 2019/20: 8 cases [10.3%], P = 1.0). Baseline characteristics were similar in patients with and without IAPA, except for a significantly higher prevalence of asthma among patients with IAPA (P = 0.05, Table 1). Patients with and without asthma received corticosteroids in 3/8 (37.5%) versus 27/150 (18%, P = 0.2) before influenza diagnosis and in 7/8 (87.5%) versus 76/150 (50.7%, P = 0.05) during hospitalization.

**TABLE 1:** Baseline characteristics

	Non‐IAPA	IAPA	P value
	n = 141 (89.2%)	n = 17 (10.8%)	
Age (years, median, (IQR))	65 (50–73)	58 (56–63)	0.24
SAPS II score (median, (IQR))	43 (32–63)	57 (35–59)	0.39
Female sex, n (%)	65 (46)	6 (35)	0.45
Influenza type A, n (%)	92 (65)	12 (71)	0.54
COPD, n (%)	43 (31)	4 (24)	0.53
**Asthma n (%)**	**5 (4)**	**3 (18)**	**0.05**
Solid organ transplant, n (%)	1 (1)	1 (6)	0.68
Hematologic malignancy, n (%)	14 (10)	1 (6)	1.0
Lymphopenia^a^, n (%)	84 (60)	13 (78)	0.20
Neutropenia^a^, n (%)	3 (3)	1 (6)	0.44
Diabetes mellitus, n (%)	28 (21)	5 (30)	0.37
Cardiovascular disease, n (%)	54 (38)	7 (41)	0.80
Obesity, n (%)	24 (17)	0	0.07
Renal failure, n (%)	40 (28)	7 (41)	0.28
Corticosteroid before influenza diagnosis, n (%)	27 (19)	4 (24)	0.75
Immunosuppressive treatment before influenza diagnosis, n (%)	22 (16)	3 (18)	0.73
Antibiotics with ICU entry n (%)	63 (45)	7 (41)	0.78

*Note*: Obesity = BMI ≥ 30 kg/m^2^, corticosteroids before influenza diagnosis ≥ 0.1 mg/kg/day prednisone equivalent.

Abbreviations: COPD, chronic obstructive pulmonary disease; IAPA, influenza‐associated pulmonary aspergillosis; ICU, intensive care unit; IQR, interquartile range; non‐IAPA, influenza patients without IAPA; SAPS II, simplified acute physiology score, predicts mortality in ICU patients.^17^

^a^ At influenza diagnosis.

### 3.1 Diagnostics

Despite our proposed algorithm, GM was more often measured (overall: 94% vs. 40%, P < 0.001) in patients with IAPA than those without IAPA (Table 2). GM was positive in serum in 57% versus 3% and in BAL in 42% versus 0% (P < 0.001) of patients with IAPA and without IAPA. Cultural growth of *Aspergillus* spp. was observed in 88% of patients with IAPA and in one non‐IAPA patient in whom BAL was performed (P < 0.001). All patients with IAPA had radiological infiltrates on chest x‐ray or computer tomography (CT)‐scan compared with 83% of non‐IAPA patients (P = 0.08). IAPA was proven in one patient with histopathological evidence of invasive aspergillosis, and all other patients were classified as probable IAPA (94%).

**TABLE 2:** IAPA diagnostics

	Non‐IAPA	IAPA	P value
	n = 141 (89.2%)	n = 17 (10.8%)	
Any respiratory sample collected, n (%)	83 (59)	17 (100)	<0.001
BAL, n (%)	38 (27)	13 (76)	<0.001
Any galactomannan measured, n (%)	57 (40)	16 (94)	<0.001
Serum, n (%)	40 (28)	14 (82)	<0.001
BAL, n (%)	33 (42)	12 (71)	0.04
Elevated galactomannan, n (%)^a^	1 (2)	8 (50)	<0.001
Serum, n (%)	1 (3)	8 (57)	<0.001
BAL, n (%)	0	5 (42)	‐
Growth of *Aspergillus* spp., n (% tested)	1 (1)	15 (88)	<0.001
Histopathological evidence of IAPA, n (%)	0	1 (6)	‐
Infiltrates on CXR/CT‐scan, n (%)	117 (83)	17 (100)	0.08

Abbreviations: BAL, bronchoalveolar lavage; CT, computer tomography; CXR, chest X‐ray; IAPA, influenza‐associated pulmonary aspergillosis; non‐IAPA, influenza patients without IAPA.

^a^ Elevated galactomannan was defined as >0.5 in serum and >1.0 in BAL.

### 3.2 Organ supportive therapies and complications

Organ supportive therapies including invasive mechanical ventilation (88% vs. 53%, P = 0.001) and vasoactive support (75% vs. 46%, P = 0.03) were required more commonly in patients with IAPA than those without (Table 3). Median duration of mechanical ventilation was significantly longer in IAPA patients (14 [interquartile range (IQR): 11, 20] days vs. 2 [IQR: 0, 9] days, P ≤ 0.001) but not extended in asthmatic patients (0 vs. 2 days in influenza patients with and without asthma and 15 vs. 11 days in IAPA patients with and without asthma, P = 0.4). Complications were common in all patients with influenza infection but more common in IAPA patients, such as acute respiratory distress syndrome^18^, ^19^ (ARDS, 53% vs. 26%, P = 0.04) and bacterial respiratory infection (65% vs. 37%, P = 0.04). IAPA patients had higher ICU‐mortality (35% vs. 16.4%, P = 0.05) and more frequently poor outcomes (94% vs. 46%, P < 0.001).

**TABLE 3:** Organ supportive therapies and complications

	Non‐IAPA	IAPA	P value
	n = 141 (89.2%)	n = 17 (10.8%)	
Organ supportive therapies			
**Invasive mechanical ventilation, n (%)**	**74 (53)**	**15 (88)**	**0.001**
**Ventilator days, median (IQR)**	**2 (0–9)**	**14 (11–20)**	**<0.001**
Renal replacement therapy, n (%)	19 (14)	5 (29)	0.14
ECMO, n (%)	18 (12)	4 (24)	0.26
**Vasoactive support, n (%)**	**64 (46)**	**13 (75)**	**0.03**
Complications			
Any complication, n (%)	97 (69)	15 (88)	0.15
**ARDS, n (%)**	**37 (26)**	**8 (53)**	**0.04**
**Respiratory bacterial infection, n (%)**	**52 (37)**	**11 (65)**	**0.04**
**ICU mortality, n (%)**	**20 (14)**	**6 (35)**	**0.05**
**Combined poor outcome, n (%)**	**65 (46)**	**16 (94)**	**<0.001**

*Note*: Combined poor outcome was defined as a composite of in‐hospital mortality, ICU length of stay ≥ 7 days, invasive mechanical ventilation ≥ 7 days, and ECMO.

Abbreviations: ARDS, acute respiratory distress syndrome according to Berlin criteria^18^, ^19^; ECMO, extracorporeal membrane oxygenation; IAPA, influenza‐associated pulmonary aspergillosis; ICU, intensive‐care unit; IQR, interquartile range; non‐IAPA, influenza patients without IAPA.

### 3.3 Predictors for IAPA and poor outcome (multivariate analysis)

**FIGURE 2:**
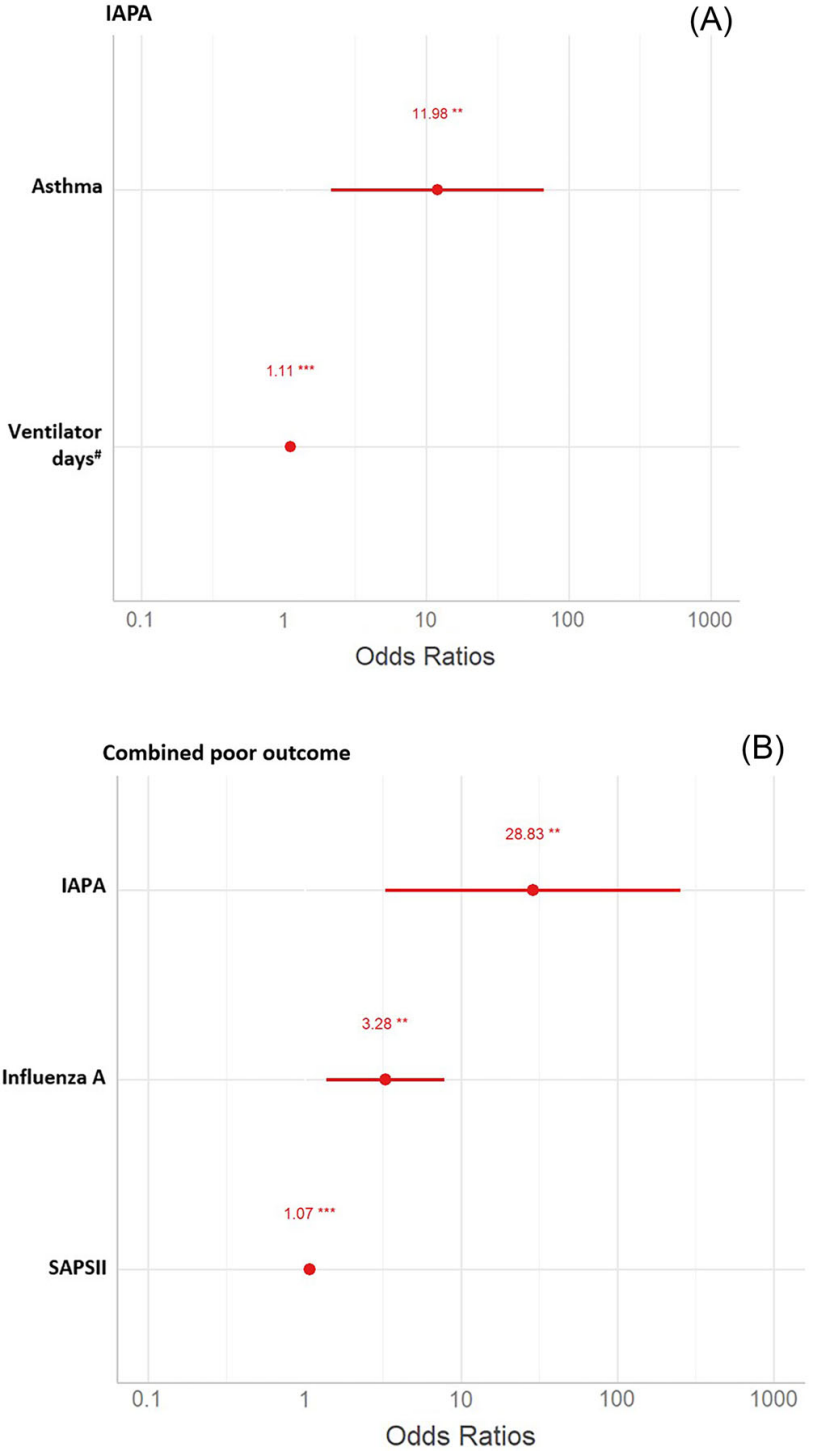
Predictors for IAPA (A) and combined poor outcome (B). Forrest plots of predictors of influenza‐associated aspergillosis (A) and combined poor outcome (B) in multivariable analysis. IAPA = influenza‐associated pulmonary aspergillosis, SAPS II = simplified acute physiology score, estimates mortality in ICU patients,^17^ *P ≤ 0.05, **P < 0.01, ***P < 0.001, ^#^odds ratio per day of mechanical ventilation

**FIGURE 3:**
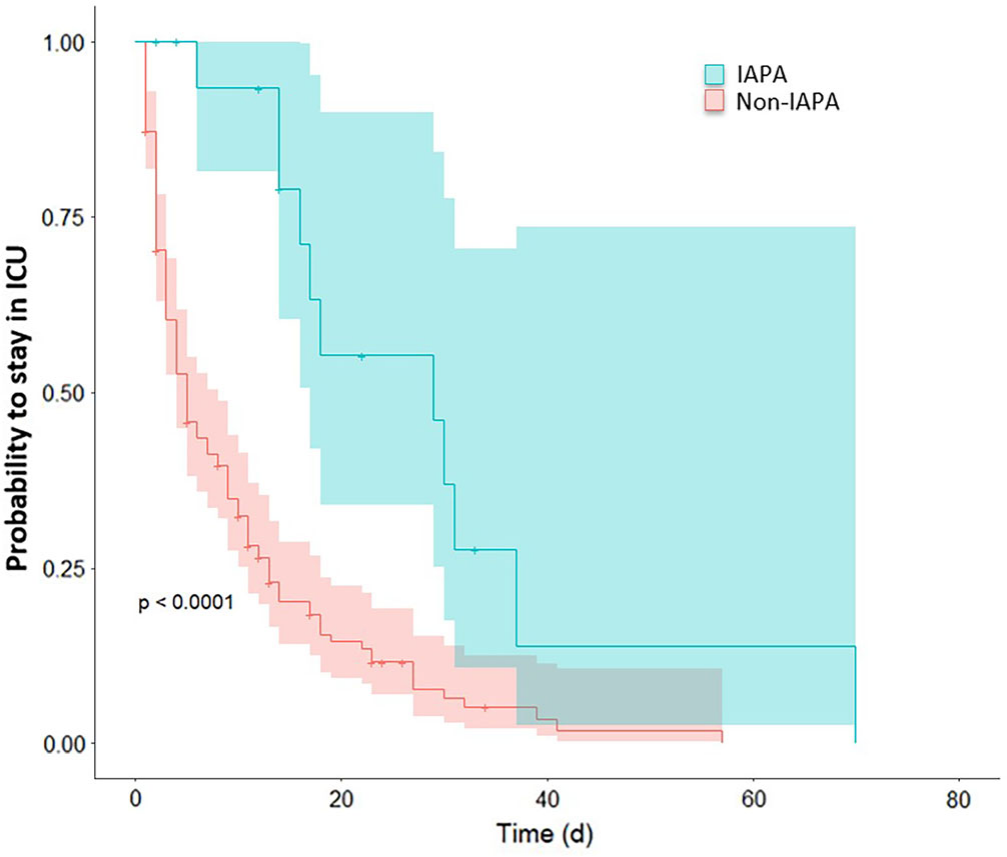
Length of ICU stay. Kaplan–Meier curve on length of ICU stay in IAPA (blue) and non‐IAPA patients (red). Probability to stay in the ICU is shown on x‐axis, time after admission to ICU are shown on y‐axis, P < 0.001. IAPA = influenza‐associated pulmonary aspergillosis, non‐IAPA = influenza infection without IAPA, ICU = intensive‐care unit

Asthma (OR 12.0 [95% confidence interval (CI) 2.1–67.2]) and days of mechanical ventilation (OR 1.1 [95% CI 1.1–1.2]) were independent predictors for IAPA (Figure 2). IAPA (OR 28.8 [95% CI 3.3–253.4]), infection with influenza A (OR 3.3 [95% CI 1.4–7.8]), and illness severity (Simplified Acute Physiology Score, SAPS II) (OR 1.1 [95% CI 1.05–1.10]) were independently associated with poor outcome (Figure 2). IAPA was associated with significantly longer median ICU‐LOS (29 [95% CI: 11–17] days vs. 5 [95% CI: 4–8] days, P < 0.001; Figure 3).

## 4 DISCUSSION

This study on critically ill influenza patients in seven tertiary care hospitals in Switzerland found an IAPA prevalence of 10.8% over two influenza seasons. IAPA was independently associated with asthma and duration of mechanical ventilation and was an independent risk factor for poor outcome. Other independent predictors of poor outcome were influenza A and higher SAPS II.

IAPA patients needed more and longer organ supportive therapies including mechanical ventilation and vasoactive support and had longer ICU‐LOS. Complications and ICU‐mortality were more frequent in IAPA.

To our knowledge, asthma was identified as an independent risk factor for IAPA for the first time. This seems biologically plausible. Viral infections like influenza can cause severe exacerbations in patients with asthma and chronic obstructive pulmonary disease (COPD).^20^, ^21^ Standard treatment of bronchial asthma consists of inhaled and/or systemic corticosteroids and inhaled bronchodilators. Prednisone treatment within 28 days prior to influenza infection has been recognized as a risk factor for developing IAPA^1^ and corticosteroid treatment is a risk factor for invasive fungal infection in lung disease.^22^ Corticosteroid treatment was also shown to cause a higher fungal burden in the lung.^23^ Therefore, application of inhaled and/or systemic corticosteroids in asthma patients could explain the higher risk for IAPA. Our data also show more frequent corticosteroid treatment in asthma patients during hospitalization. In addition, asthma patients have altered mucociliar clearance of the lung that explains higher rates of fungal growth and colonization in these patients.^24^ High clinical suspicion, early and regular screening for IAPA are therefore warranted in asthma patients. Similarly, this underlines the importance of influenza vaccination for patients with asthma as recommended in Swiss guidelines.^25^

The colonization with *Aspergillus* spp.—a prerequisite for the development of IAPA—likely occurs prior to ICU admission as suggested by the POSA‐flu trial, where posaconazole prophylaxis started on ICU admission had failed to prevent IAPA or lower mortality in influenza patients.^15^ The authors therefore concluded that development of invasive fungal disease occurs early after influenza infection as 71% of IAPA cases were diagnosed within 24 h of ICU admission. This seems plausible since colonization with *Aspergillus* spp. is a known risk factor for developing invasive aspergillosis.^26^ Identification of asthma as a risk factor for IAPA further strengthens this pathophysiologic hypothesis since asthma patients are frequently colonized with *Aspergillus* spp.^24^, ^27^

This study identified IAPA, high SAPS II, and infection with influenza A as predictors for poor outcome in critically ill patients with influenza. High mortality in patients with IAPA has been reported by us and others.^1^, ^14^ Interestingly, influenza A was associated with poor outcome that was also shown in a recent meta‐analysis of 14 studies of IAPA.^28^ An association of influenza A with an increased risk of bacterial respiratory infections and mortality has been previously suggested,^29^, ^30^, ^31^ even though it was recently challenged.^32^

The proportion of IAPA among influenza patients requiring ICU care in Switzerland was similar in the 2017/2018 and the 2019/2020 seasons,^14^ which were characterized by influenza B with Yamagata (2017/2018) predominance versus similar presence of influenza A(H1N1)pdm09 and B Victoria (2019/2020). This is in line with previous reports^1^, ^12^ suggesting that IAPA is not restricted to a selected influenza seasons.^1^

This study is limited by its retrospective design. To optimize pre‐analytics and screening of IAPA in influenza patients, a screening algorithm was installed at the beginning of the influenza season 2019/2020 in all participating ICUs. Despite the recommended screening algorithm for IAPA, sampling of respiratory material and testing for GM was underutilized. This could have resulted in an underestimation of IAPA diagnosis and growth of *Aspergillus* spp. in respiratory samples in the non‐IAPA group. However, growth of *Aspergillus* spp. was only observed in one patient in the non‐IAPA group in which 59% had at least one respiratory sample taken. We therefore assume that most IAPA patients were correctly classified and identified and results can be generalized to critically‐ill influenza patients. Because the setting was ICU‐specific no conclusions can be made regarding IAPA in patients in an ambulatory setting or hospitalized on the ward. Also, generalizability of results is limited by small numbers of IAPA patients. The observation of a higher risk of IAPA in asthmatic patients does not prove causality and should be confirmed in larger preferably prospective cohort studies.

## 5 INTERPRETATION

In conclusion, our data stress the importance of diagnosing IAPA in patients with influenza in the ICU. IAPA was a frequent complication of influenza with high associated mortality, frequent need of organ supportive therapies and longer stay in the ICU. Furthermore, asthma was newly identified as a risk factor for IAPA. We call for increased awareness of IAPA in critically ill asthma patients with influenza, including more intense screening strategies. Prevention efforts through influenza vaccination should be improved in asthma patients as well.

## CONFLICT OF INTEREST

WCA: Honoraria for presentations for A. Vogel and Pfizer and advisory boards for GSK, MSD, OM Pharma, Pfizer, Sanofi. Reimbursements were paid to his institution.

## AUTHOR CONTRIBUTIONS

**Filippo Boroli:** Conceptualization; data curation; investigation; validation. **Sandra Zingg:** Data curation; formal analysis; investigation; validation. **Laura N. Walti:** Data curation; formal analysis; investigation; validation. **Pedro David Wendel‐Garcia:** Data curation; formal analysis; investigation; validation. **Anna Conen:** Data curation; formal analysis; investigation; validation. **Jean‐Luc Pagani:** Data curation; formal analysis; methodology; validation. **Katia Boggian:** Supervision; validation. **Madeleine Schnorf:** Data curation; investigation. **Martin Siegemund:** Data curation; formal analysis; investigation; validation. **Samia Abed‐Maillard:** Investigation; project administration. **Marc Michot:** Data curation; investigation; validation. **Yok‐Ai Que:** Data curation; formal analysis; investigation; validation. **Veronika Bättig:** Data curation; formal analysis; investigation; validation. **Noémie Suh:** Data curation; investigation; project administration; validation. **Gian‐Reto Kleger:** Data curation; formal analysis; methodology; software; visualization. **Werner C. Albrich:** Conceptualization; formal analysis; investigation; methodology; supervision; validation.

### PEER REVIEW

The peer review history for this article is available at https://publons.com/publon/10.1111/irv.13059.

## Data Availability

Data are available upon request.
